# Supplementary material for: C1orf106 (INAVA) Is a SMAD3-Dependent TGF-β Target Gene That Promotes Clonogenicity and Correlates with Poor Prognosis in Breast Cancer
Source: Cells. 2024 Sep 12;13(18):1530. doi: 10.3390/cells13181530 (PMC11429573; doi:10.3390/cells13181530)
Supplement: Supplementary file 1 [file cells-13-01530-s001.zip › cells-2909030-supplementary.pdf]

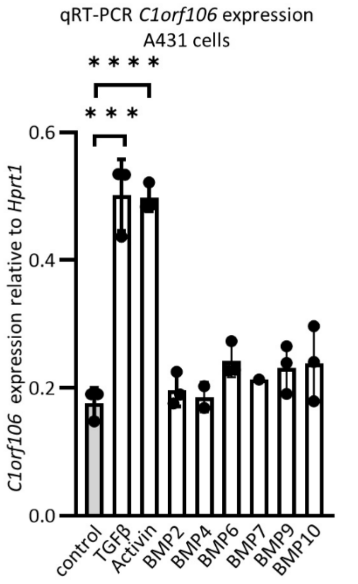

Figure S1. *C1orf106 is a common TGF-β and Activin target gene.*
A431 cells treated with a panel of TGF-β superfamily ligands (5 ng/ml TGF-β, 50 ng/ml Activin-A and 4 ng/ml of the indicated BMPs) were analysed for *C1orf106* mRNA expression after 2 hours by qRT-PCR (n=3 biological replicates.). *C1orf106* Ct values were normalised to *HPRT1*. Error bars show standard deviation. Statistical significance was determined by unpaired student's t-test: *** = p<0.001; **** = p<0.0001.

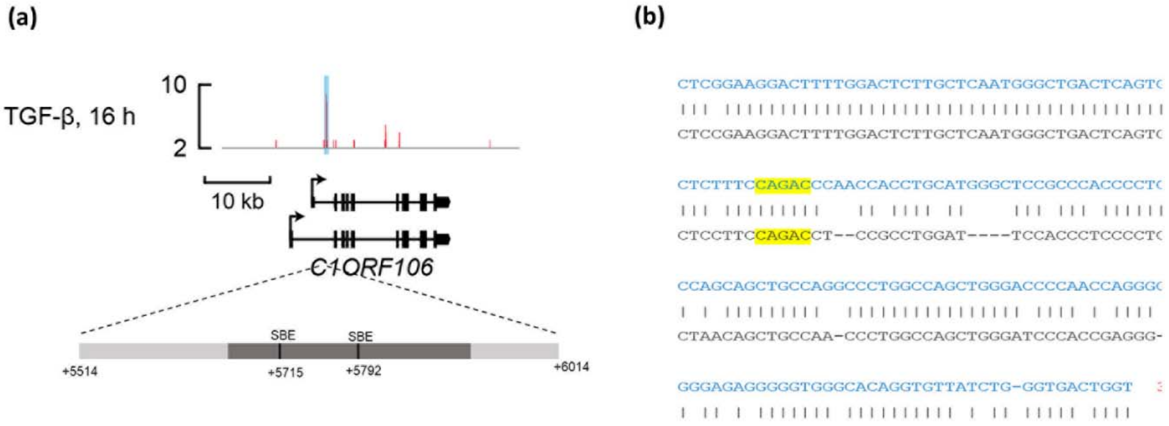

Figure S2. *Human C1orf106 intron 1 contains a TGF-β inducible SMAD2/3 binding site and this is conserved in murine C1orf106.*
(a) SMAD2/3 ChIP-seq analysis of the *C1orf106* gene locus from the dataset GSE83788. Significant SMAD2/3 binding enrichment is indicated in blue. BLAST was carried out of the identified region of SMAD2/3 binding within *C1orf106* intron 1 between human and mouse nucleotide sequences and regions of similarity within this are highlighted along with genomic coordinates according to the GRCh38.p12 and GRCm38.p6 assemblies. **(b)** Nucleotide sequence alignment of conserved region between human (top) and murine (bottom) *C1orf106* shown in (a). The potential SMAD binding element is highlighted in yellow.

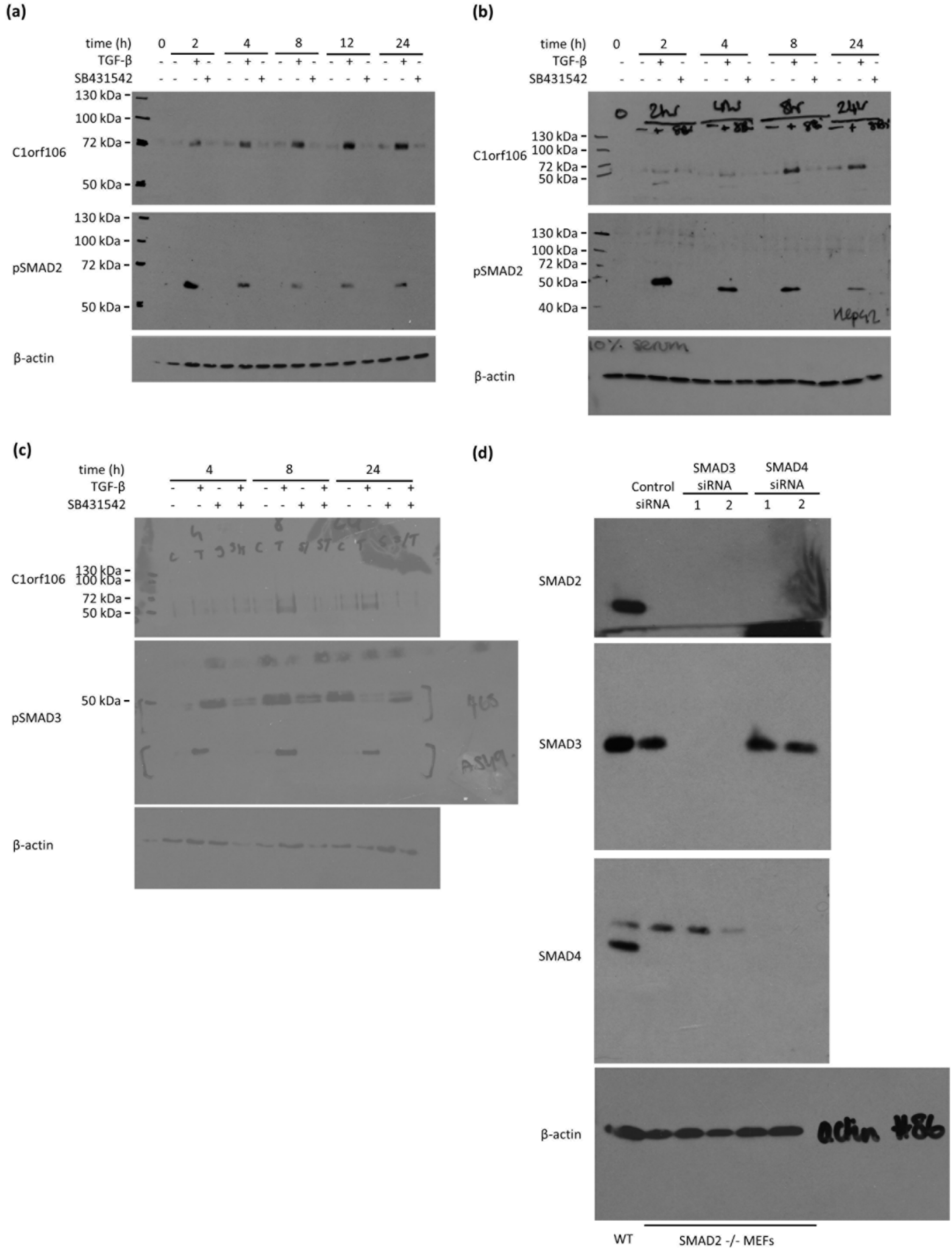

Figure S3. *Uncropped blots for Figure 1 and 2.*
(a) Raw blots for Figure 1b. **(b)** Raw blots for Figure 1c. **(c)** Raw blots for Figure 1d. **(d)** Raw blots for Figure 2b.

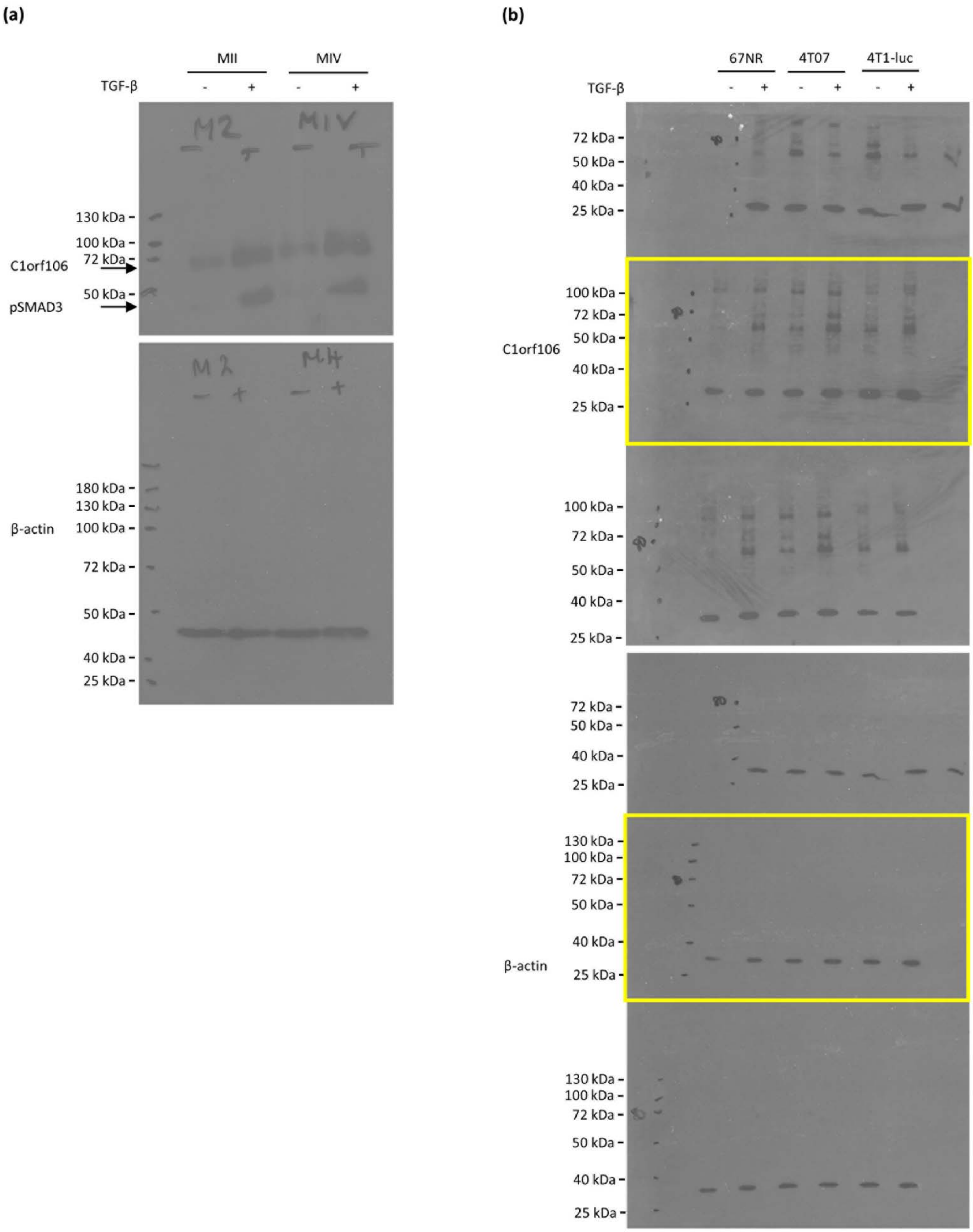

Figure S4. *Uncropped blots for Figure 3.*

(a) Raw blots for Figure 3b. **(b)** Raw blots for Figure 3d. The representative blot is marked with a yellow rectangle.

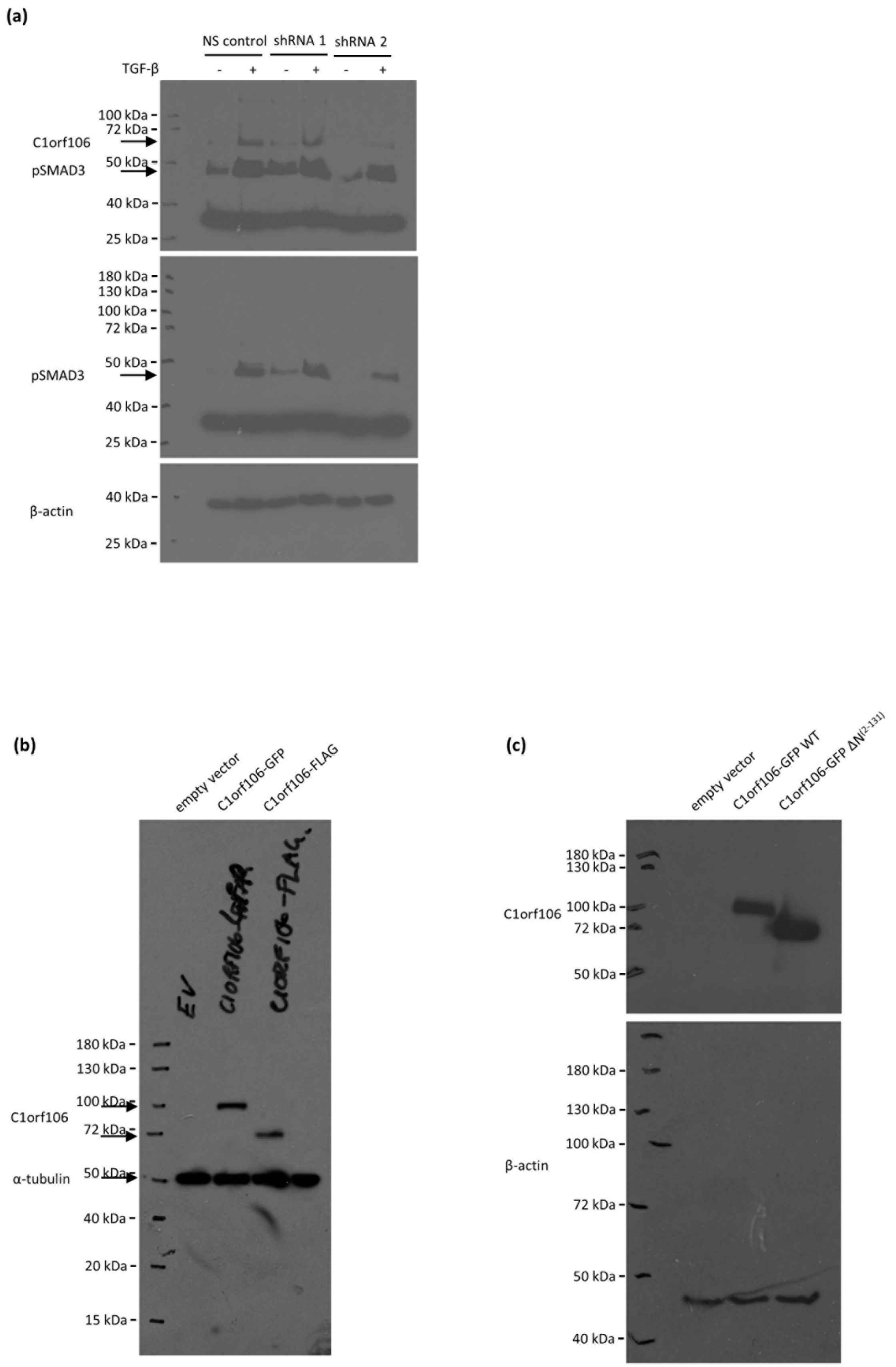

Figure S5. *Uncropped blots for Figure 3f and 5.*

(a) Raw blots for Figure 3f. **(b)** Raw blots for Figure a. **(c)** Raw5 blots for Figure 5e.

Supplementary Table S1: Orthotopic tumour development in CD1-Nude mice.

	**67NR/EV control**	**67NR/c1orf106**
	#tumour/#transplanted	#tumour/#transplanted
100 cells transplanted	9/10	9/10
50 cells transplanted	8/10	8/10
10 cells transplanted	5/10	8/10
